# Chitooligosaccharide Seed Priming Enhances Photosynthetic Efficiency in Pea (*Pisum sativum*) Under Salinity

**DOI:** 10.3390/ijms27104498

**Published:** 2026-05-18

**Authors:** Sashka Krumova, Svetozar Stoichev, Daniel Ilkov, Georgi Rashkov, Anelia Dobrikova, Emilia Apostolova, Velichka Strijkova, Vesela Katrova, Tsonko Tsonev, Violeta Velikova

**Affiliations:** 1 Institute of Biophysics and Biomedical Engineering, Bulgarian Academy of Sciences, 1113 Sofia, Bulgaria; sashka.b.krumova@gmail.com (S.K.); thylakoid85@gmail.com (S.S.); megajorko@abv.bg (G.R.); aneli@bio21.bas.bg (A.D.); emilia.apostolova@gmail.com (E.A.); 2 Institute of Plant Physiology and Genetics, Bulgarian Academy of Sciences, 1113 Sofia, Bulgaria; d_ilkov@bio21.bas.bg (D.I.); tsonev@gmail.com (T.T.); 3 Institute of Optical Materials and Technologies “Acad. Jordan Malinowski”, Bulgarian Academy of Sciences, 1113 Sofia, Bulgaria; vily@iomt.bas.bg (V.S.); vlozanova@iomt.bas.bg (V.K.)

**Keywords:** chitosan, chitosan oligosaccharides, salt stress, germination, plant growth, photosynthesis

## Abstract

Low-molecular-weight chitosan derivatives emerged as promising plant growth biostimulants due to their favorable properties, such as biocompatibility, antibacterial and antifungal activity, enhancement of stress resistance, and yield improvement. In the present study, we evaluated the effect of pea seed priming with two types of chitooligosaccarides (aminochitooligosaccaride and chitooligosaccaride hydrochloride) applied at concentrations of 100 and 500 mg/L under non-stress conditions and 50 mM chronic NaCl stress. We characterized the seed surface topology by atomic force microscopy, the germination process by evaluation of seed germinability and synchrony, root emergence, seed imbibition capacity and ion leakage. Early plant growth and physiological performance were further evaluated in 14-day-old seedlings by measuring leaf water potential, Na^+^ accumulation in roots and leaves, photosystem II activity, leaf pigment content, and membrane stability. The results revealed changes in seed coat topology, i.e., higher surface roughness in 100 and 500 mg/L chitooligosaccaride hydrochloride and 500 mg/L aminochitooligosaccaride primed variants. Concentration-dependent effects of the two chitooligosaccarides under both non-stress and salt stress conditions were evident in 14-day-old seedlings. Under chronic salt stress, seed priming with 100 mg/L chitooligosaccharide hydrochloride and 500 mg/L aminochitooligosaccharide produced the most pronounced improvements in the primary photochemical reactions of photosynthesis, particularly the performance index on an absorption basis and the total performance index. Moreover, the investigated chitooligosaccharide, particularly chitooligosaccaride hydrochloride, preserved membrane integrity and maintained flavonol and anthocyanin levels, indicating a strong protective effect against salt stress. Overall, the data indicate beneficial effects on pea physiological status following seed priming with chitooligosaccarides under chronic salt stress conditions. This highlights the approach as a promising strategy for enhancing plant resilience in challenging environments, and it is worth further investigation and verification at the whole-plant level.

## 1. Introduction

Salt stress is among the most common stresses that plants experience, both in coastal regions and continental areas with prolonged drought periods. According to the Food and Agriculture Organization of the United Nations, up to 1.5 million hectares of arable land are lost annually due to increasing soil salinity [1]. The detrimental effects of salinity on plant growth arise from a combination of osmotic stress and ionic toxicity due to Na^+^ and Cl^−^ accumulation [2]. These factors lead to ion imbalance, membrane damage, reactive oxygen species (ROS) accumulation, and metabolic and photosynthetic abnormalities, which consequently impair plant growth [3]. The photosynthetic apparatus is among the main targets of salt stress in plants. Salination affects the number and ultrastructure of chloroplasts with respect to plastoglobules and grana formation, pigment composition and structural organization of photosynthetic pigment-protein complexes, as well as lipid metabolism and peroxidation [4,5,6,7,8,9]. These structural changes result in functional impairment of photosystem II (PSII) and the associated oxygen-evolving complex (OEC), and consequently the whole photosynthetic electron-transport chain [10,11,12,13,14]. Plants protect photosystems via non-photochemical quenching, cyclic electron flow, and activation of antioxidant defenses, preserving redox balance and photosynthetic performance [15,16]. Changes in chlorophyll content further modulate light-harvesting capacity, while anthocyanins and flavonoids act as non-enzymatic antioxidants, scavenging ROS, stabilizing membranes, and filtering excess light [17,18,19]. Their accumulation under salt stress is often species- and context-specific, yet essential for protecting electron transport and maintaining PSII functionality.

The large economic impact of salt stress worldwide triggers the development of diverse agronomic strategies to combat acute and persistent salinity. Different approaches and strategies are being developed to optimize photosynthesis, mainly relying on genetic modifications [20] whose main targets are the photophysical, photochemical or biochemical reactions of photosynthesis [21]. Photophysical reactions include the phase of light capture (with the help of specific pigments, mainly chlorophylls and carotenoids) and the transfer of excitation energy to various scattering paths, including the reaction centers (RC) of photosystems. The photochemical reactions follow the photophysical ones and include the splitting water (photolysis), electron transfer, proton transfer, and the formation of ATP and NADPH, which are then used in subsequent biochemical reactions in the Calvin−Benson cycle. Due to the complexity of photosynthetic reactions, often the modification of just one component of the photosynthetic apparatus can cause a “cascade” of changes with unknown consequences. Moreover, this often requires multiple gene transformation, which may not be feasible, at least in short times. Over the past two decades, interest in organically grown crops as well as the application of naturally driven plant biostimulants has been on the rise worldwide. Several studies have documented the benefits of plant biostimulant application on growth, crop productivity and quality, as well as on stress tolerance [22,23]. Among them, supplementation with chitosan and its derivatives stems as a promising novel ecological approach [24]. However, detailed information about the potential benefits from applications of plant biostimulants on the structure and functionality of the photosynthetic apparatus is very limited.

Chitooligosaccharides (COS) are considered a more beneficial alternative to chitosan. Several reports have demonstrated that these low-molecular-weight derivatives are more soluble than chitosan while preserving or even enhancing its beneficial properties, including biocompatibility, antibacterial and antifungal activity, resistance to abiotic and biotic stresses, and yield enhancement [25,26]. Furthermore, they positively affect photosynthesis, including under conditions of seed exposure to salt stress. An et al. [27] showed that when applied via soil supplementation under conditions of salt stress, COS induce a reduction in Na^+^ content, but an increase in leaf Ca^2+^ level, fresh biomass, transpiration rate, chlorophyll content, stomatal conductance and photosynthetic activity of cotton seedlings. Wheat seed soaking with COS exerts a protective effect against NaCl-induced stress at the stage of fully expanded seedlings, in terms of shoot and root length, chlorophyll content, photosynthetic rate, stomatal conductance, and antioxidant defense [28].

For decades, seed priming was utilized to enhance seed resistance and growth [29,30]. It relies on pre-soaking in water/solution for a certain period (typically reaching a lag phase in seed weigh increase) followed by a natural drying step to the initial seed weight and consequent rehydration before sowing [31]. During this process, the seeds are kept in a pre-germination state, allowing for the rapid resumption of metabolic activity. Several compounds, including inorganic salts, β-aminobutyric acid and plant hormones, have been identified as effective priming agents under salt stress conditions [32]. Chitosan seed priming was also reported to help combat salt stress [33], whereas the use of low-molecular-weight COS for this purpose remains unexplored. Moreover, to the best of our knowledge, no studies have yet addressed the effects of COS on seed coat structure or provided a detailed characterization of the photosynthetic electron transport chain, both of which may influence seed and photosynthetic performance under physiological and stress conditions. Therefore, the present study aimed to evaluate the effects of COS seed priming on plant physiological traits in pea plants under non-stress and salt stress conditions. To address this, two derivatives of COS (aminooligochitosan, NH_2_-COS and chitosan hydrochloride, HCl-COS) were applied in concentrations of 100 and 500 mg/L, and their effects on seed germination and seedling fitness were assessed under 50 mM NaCl stress in comparison with non-stress conditions.

By combining physiological measurements, chlorophyll *a* fluorescence induction kinetics OJIP curves analysis, and structural characterization of seed surface by atomic force microscopy (AFM), the present study provides mechanistic insights into COS-mediated priming effects, highlighting their potential to enhance plant resilience under saline conditions.

## 2. Results

Two types of COS, aminooligochitosan (denoted hereafter as NH_2_-COS_100/500_ for concentrations 100 and 500 mg/L, respectively) and chitosan hydrochloride (denoted hereafter as HCl-COS_100/500_ for concentrations 100 and 500 mg/L, respectively), were evaluated for their priming effects on a panel of growth and photosynthetic parameters. Water-soaked seeds served as the H_2_O-primed control.

### 2.1. Effect of COS-Priming on Seed Integrity and Surface Topology

**Figure 1:**
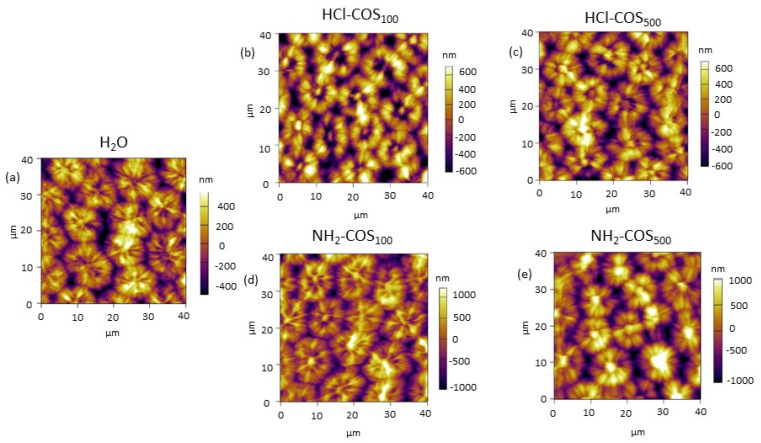
Representative AFM images of the seed topology of H_2_O- (**a**) and COS-primed (**b**–**e**) variants.

AFM was first utilized to probe the effect of the selected COS on the surface structure of pea seeds. As can be seen from Figure 1, all variants (H_2_O- and COS-primed) exhibited the typical surface topology for pea seeds, defined by the arrangement of macrosclereids into circular formations. The height of the formations differed among the various priming treatments (600–800 nm for H_2_O-priming, 800–1500 nm for HCl-COS-priming and 800–2000 nm for NH_2_-COS-priming), which was reflected in the estimated roughness (R_rms_) values. The data revealed that the roughness of the HCl-COS_100_ (404 ± 22), HCl-COS_500_ (339 ± 30), and NH_2_-COS_500_ (421 ± 18) variants significantly exceeded that of the H_2_O-primed (266 ± 31) seeds, while NH_2_-COS_100_ (264 ± 30) retained values that did not differ significantly from the control (Table 1).

Seed imbibition capacity and ion leakage from the seed coat were also assessed to determine the effects of treatments on water uptake and seed coat integrity, respectively. Imbibition percentage varied in the range of 227–239% (i.e., about 5% variation). The conductivity values remained in the range of 828–978 µS/cm^2^ (i.e., about 18% variation) for all treatments (Figure S1). Thus, no significant differences were observed among the different priming treatments, indicating that COS application does not exert any negative impact on seed quality, i.e., it does not impair the uptake of water-soluble substances from the environment, and it does not compromise the seed coat integrity (Figure S1).

### 2.2. Effect of COS-Priming on Seed Germination Uunder Control and Salt Stress Conditions

The germination process and the consequent plant growth of the different seed treatments were compared under control and salt stress conditions. To mimic chronic salt stress in the environment, seeds and plants were grown in salt-supplemented medium from the onset of germination to the end of the experiment (i.e., 14-day-old seedlings).

As shown in Figure S2, the germination rates of seeds were high under control (non-stress) conditions (87–95%). Upon salting, the germinability of the H_2_O-primed variant dropped insignificantly (by ≈6%), and no statistically significant differences were detected among the different COS-primed variants. Salt stress exposure did not influence seed germination synchrony in any of the investigated treatments (Figure S2).

**Figure 2:**
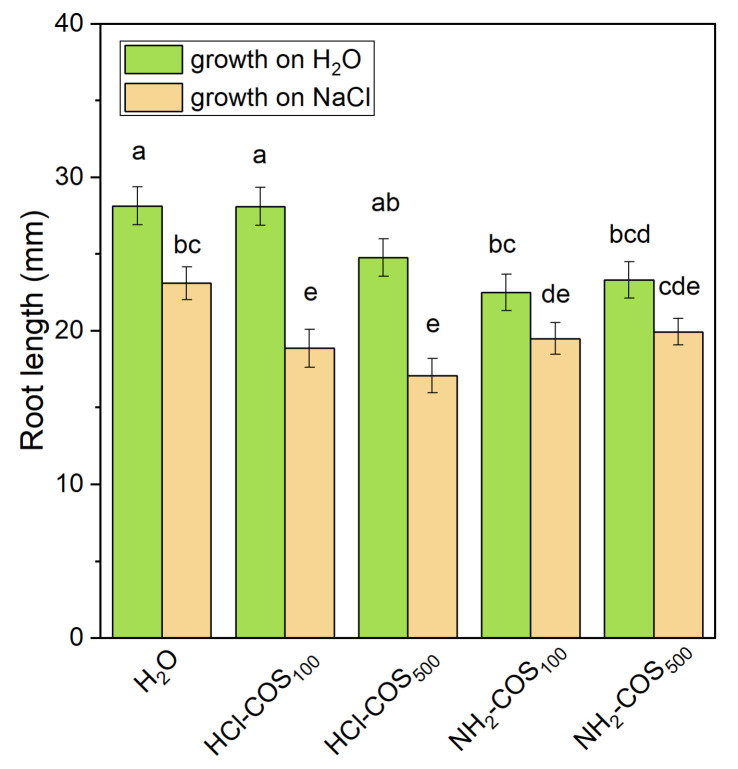
Root length of 4-day-old seedlings derived from H_2_O- and COS-primed seeds, grown under control conditions or under 50 mM NaCl stress. Values are expressed as means ± SE (*n* = 53−63). Means with no letters in common differ significantly according to the Duncan’s multiple range test (*p* < 0.05).

The root length measured on the fourth day of germination (see Figure S3) was approximately 28 mm in the H_2_O-primed variant under non-stress conditions. It did not change significantly in the HCl-COS-treated variants but was reduced by about 21% in both NH_2_-COS variants. Under salt stress, the root length in H_2_O-primed seeds decreased by approximately 18% compared with the non-stress control. In contrast, all COS-treated variants exhibited a more pronounced reduction in root length (14–26%) relative to the salt-stressed control (Figure 2).

### 2.3. Effect of COS-Priming on the Water Potential and Na^+^ Content in Pea Seedlings Under Control and Salt Stress Conditions

To assess the effects of COS pea seed priming on plant salt stress tolerance, we measured leaf water potential and Na^+^ accumulation in leaves and roots in non-stressed and salt-stressed seedlings.

**Figure 3:**
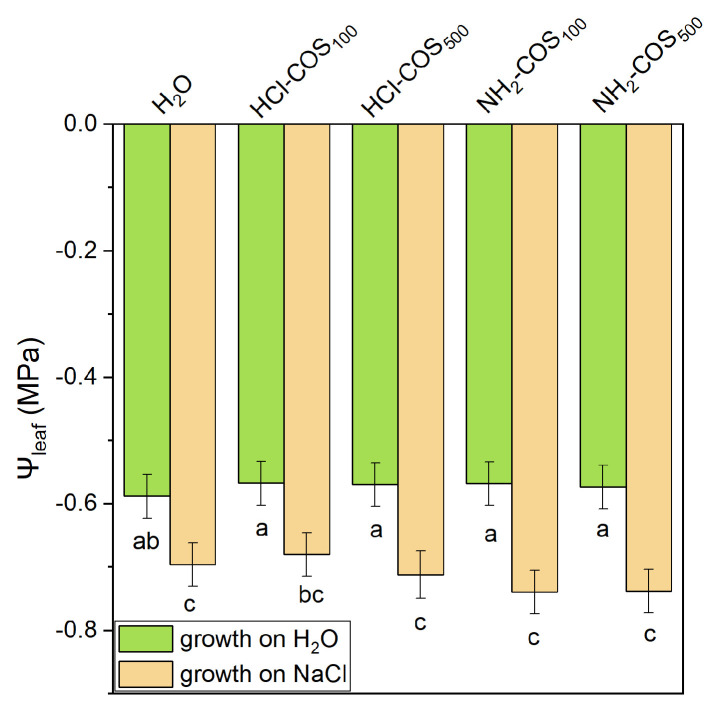
Leaf water potential (Ψ_leaf_) of 14-day-old seedlings derived from H_2_O- and COS-primed seeds, grown under non-stress conditions or under 50 mM NaCl stress. Values are expressed as means ± SE (*n* = 6). Means with no letters in common differ significantly according to the Duncan’s multiple range test (*p* < 0.05).

Under control conditions, leaf water potential (Ψ_leaf_) in COS-primed variants was comparable to that of the H_2_O-primed control. Under salt stress, Ψ_leaf_ decreased in all treatments, i.e., by 17% in the H_2_O-primed variant and by 29% in COS-primed plants, with insignificant variation among the COS-primed variants (Figure 3).

**Figure 4:**
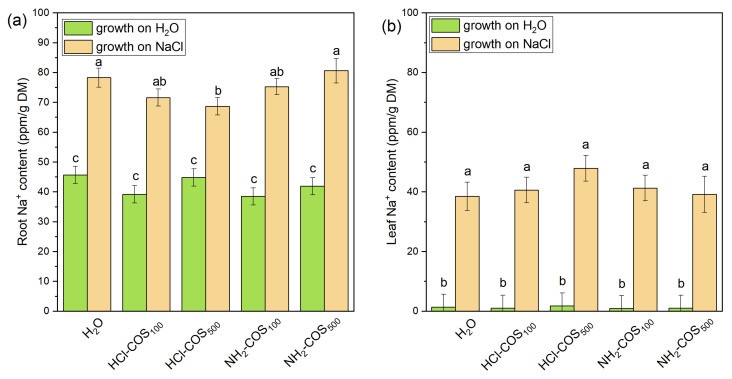
Na^+^ content in roots (**a**) and leaves (**b**) of 14-day-old seedlings derived from H_2_O- and COS-primed seeds, grown under control conditions or under 50 mM NaCl stress. Values are expressed as means ± SE (*n* = 6). Means with no letters in common differ significantly according to the Duncan’s multiple range test (*p* < 0.05).

Under non-stress conditions, Na^+^ accumulation in H_2_O- and COS-primed variants ranged from 38.5 to 45.7 ppm/g dry mass (DM) in roots and from 1.05 to 1.83 ppm/g DM in leaves (Figure 4), with no statistically significant differences among treatments. Under salt stress, Na^+^ levels in the H_2_O-primed variant increased by ≈1.7-fold in roots and ≈28-fold in leaves, compared to the non-stressed control. No statistically significant differences in Na^+^ accumulation in roots and leaves were observed between COS-treated and H_2_O-primed variants under salt stress, except for the HCl-COS_500_ treatment, which showed a 12% lower Na^+^ content in roots relative to the H_2_O-primed variant (Figure 4).

### 2.4. Effect of COS-Priming on the Photosynthetic Activity of Pea Seedlings Under Control and Salt Stress Conditions

To evaluate the functional state of the photosynthetic apparatus, we analyzed chlorophyll *a* fluorescence induction (OJIP transitions). From the recorded OJIP curves (Figure S4), we calculated the following parameters [34,35]: the ratio of quantum yields of photochemical and concurrent non-photochemical processes (Fv/Fo); the relative variable fluorescence at the J step (Vj), indicating the proportion of closed PSII reaction centers; the efficiency of reduction in the end electron acceptors at the photosystem I (PSI) acceptor side (δRo); the density of the active reaction centers (RC/ABS); the quantum yield of electron transport beyond Q_A_^−^ (φEo) and quantum yield of energy dissipation in the form of heat and fluorescence at the reaction center level (DIo/RC); the performance index on an absorption basis (PI_ABS_) and the total performance index (PI_total_). The PI_ABS_ index includes three parameters: the number of active PSII RC per antenna chlorophyll [γRC2/(1 − γRC2) = RC/ABS], the partial performance of primary photochemistry [φPo/(1 − φPo)], and the performance of thermal reactions of the intersystem electron carriers [ψ(Eo)/(1 − (ψEo))]. The PI_total_ index has an additional component [δ(Ro)/(1 − (δRo))], which characterizes the probability with which an electron is transferred to end PSI electron acceptors.

**Figure 5:**
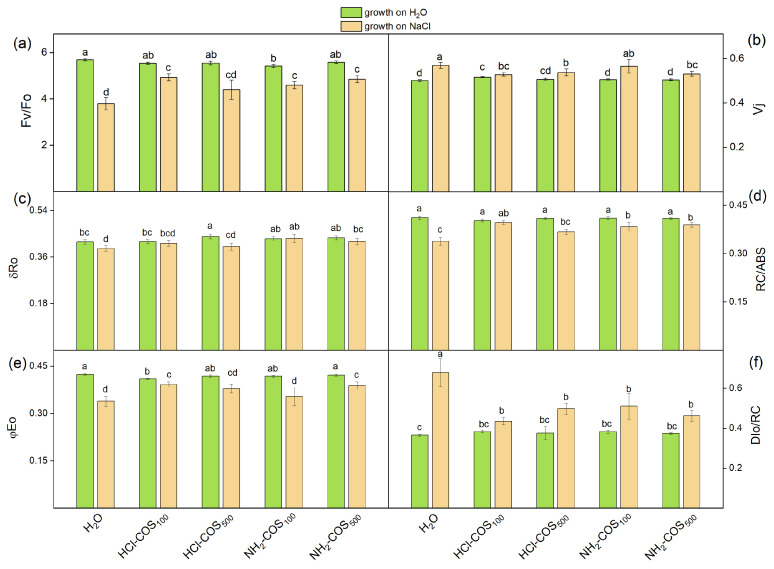
Selected JIP parameters determined on leaves of 14-day-old seedlings derived from H_2_O- and COS-primed variants grown in H_2_O or 50 mM NaCl: (**a**) ratio of quantum yields of photochemical and concurrent non-photochemical processes (Fv/Fo); (**b**) relative variable fluorescence at the J step (Vj); (**c**) efficiency with which an electron from the intersystem electron carriers is transferred to reduce end electron acceptors at the PSI acceptor side (δRo); (**d**) density of the active reaction centers (RC/ABS); (**e**) quantum yield of electron transport beyond Q_A_^−^ (φEo); (**f**) quantum yield of energy dissipation in the form of heat and fluorescence at the reaction center level (DIo/RC). Values are expressed as means ± SE (*n* = 20). Means with no letters in common differ significantly according to the Duncan’s multiple range test (*p* < 0.05).

**Figure 6:**
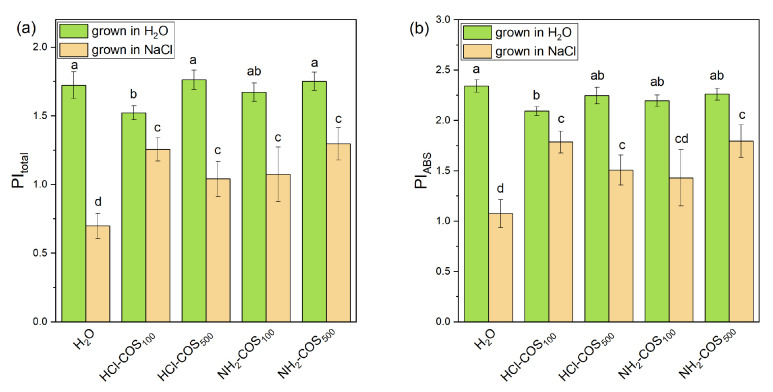
Performance indices PI_total_ (**a**) and PI_ABS_ (**b**), calculated from OJIP curves recorded for leaves of 14-day-old seedlings derived from H_2_O- and COS-primed variants grown in H_2_O or 50 mM NaCl. Values are expressed as means ± SE (*n* = 20). Means with no letters in common differ significantly according to the Duncan’s multiple range test (*p* < 0.05).

The values of the calculated JIP parameters obtained from leaves of non-treated and salt-treated plants developed from COS-primed seeds are shown in Figure 5 and Figure 6. The components defining PI_ABS_ and PI_total_ were also determined (Table 2).

Under non-stress conditions, COS-priming induced only minor changes in the photosynthetic parameters reported in Figure 5, with values varying within 5% of those recorded for the H_2_O-primed control. The evaluation of PI_ABS_ and PI_total_ indices revealed a reduction by 10% and 12%, respectively, only in HCl-COS_100_ treatment compared to the H_2_O-primed variant, while the other variants remained close to the control values (Figure 6). As shown in Table 2, this slight inhibitory effect of HCl-COS_100_ under non-stress conditions is associated with a small decrease in the efficiency of the primary photochemical reactions [φ(Po)/(1 − φ (Po)] by ≈3% and in the efficiency of the dark reactions of the intersystem electron carriers in photosynthesis [ψ(Eo)/(1 − ψ(Eo)] by ≈6%.

Under NaCl stress, all evaluated photosynthetic parameters in the H_2_O-primed variant deviated markedly from those observed under non-stress conditions. The most pronounced effects included a decrease in Fv/Fo (by ≈33%), φEo (by 20%) and RC/ABS (by 19%), and an increase in DIo/RC by 86% (Figure 5). Consequently, the performance indices were strongly affected, with PI_ABS_ decreasing by more than 50% and PI_total_ by nearly 60% compared with the non-stressed H_2_O-primed variant (Figure 6).

Under salt stress, COS-primed variants exhibited distinct response patterns for the different photosynthetic parameters. The Vj parameter showed only minor changes (up to 8% decrease), while δRo increased by up to 11% relative to the H_2_O-primed variant. In contrast, several photosynthetic parameters were significantly affected by COS-priming. Specifically, Fv/Fo increased by 19–29% in HCl-COS_100_, NH_2_-COS_100,_ and NH_2_-COS_500_ treatments_._ The quantum yield of electron transport beyond Q_A_^−^ (φEo) increased by ≈16% in HCl-COS_100_ and NH_2_-COS_500_ variants. The density of the active reaction centers (RC/ABS) increased by 16–20% in HCl-COS_100_, NH_2_-COS_100,_ and NH_2_-COS_500_, while the energy dissipation parameter (DIo/RC) strongly declined by 25–27% for all COS variants (Figure 5). Consistent with these changes, COS-priming significantly improved the PI_ABS_ and PI_total_ indices under salt stress by 42–65% and 49–80%, respectively, compared to the H_2_O-primed variant, except for PI_ABS_ in NH_2_-COS_100_, where the effect was not statistically significant (Figure 6). All COS-primed variants exhibited 12–30% higher values of the φ(Po)/((1 − φ(Po)) component compared with the H_2_O-primed stressed plants. For the remaining components of PI_ABS_ and PI_total_, the effects varied in significance. Nevertheless, in HCl-COS_100_ and NH_2_-COS_500_ variants, all components PI_ABS_ and PI_total_ were significantly higher than in the H_2_O-primed variant (Table 2).

### 2.5. Effect of COS-Priming on the Leaf Pigment Content Under Control and Salt Stress Conditions

**Figure 7:**
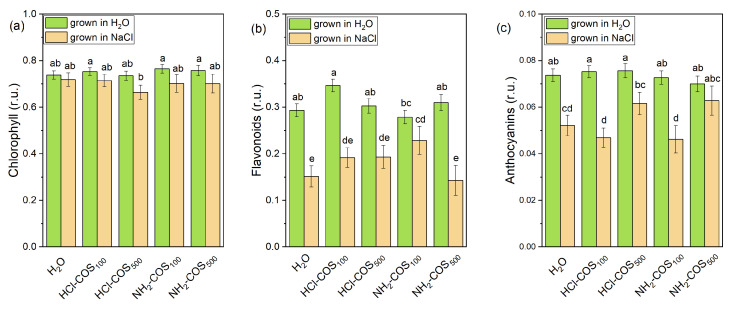
Leaf chlorophyll (**a**), flavonoids (**b**) and anthocyanin (**c**) content of 14-day-old seedlings derived from H_2_O- and COS-primed variants grown in H_2_O or 50 mM NaCl. Values are expressed as means ± SE (*n* = 23–29 for non-stress conditions and *n* = 7–15 for salt stress). Means with no letters in common differ significantly according to the Duncan’s multiple range test (*p* < 0.05).

Under non-stress conditions, the total chlorophyll, flavonoid and anthocyanin contents in plants developed from COS-primed pea seeds varied in a narrow range (within 4%, 19%, and 5% from the H_2_O-primed variant, respectively) and remained close to the control values (Figure 7).

Salt stress reduced the mean chlorophyll content of the H_2_O-treated variant by only 2%, but markedly decreased the flavonoid and anthocyanin levels by 48% and 30%, respectively (Figure 7).

Among the COS-primed variants subjected to salt stress, only a slight (7%) and not statistically significant decrease in chlorophyll content was observed in the HCl-COS_500_ treatment. Notably, only the NH_2_-COS_100_ variant exhibited significantly higher flavonoid levels (by ≈54%) compared with the H_2_O-primed stressed plants, reaching values comparable to those under non-stress conditions. Anthocyanin content was higher in plants primed with 500 mg/L compared with 100 mg/L for both HCl-COS and NH_2_-COS treatments under salt stress. In particular, the NH_2_-COS_500_ variant maintained anthocyanin levels (0.063 ± 0.006 r.u.) close to those observed under non-stress conditions (0.070 ± 0.003 r.u.), indicating a strong protective effect of this treatment (Figure 7).

### 2.6. Effect of COS-Priming on Plants Membrane Integrity Under Control and Salt Stress Conditions

**Figure 8:**
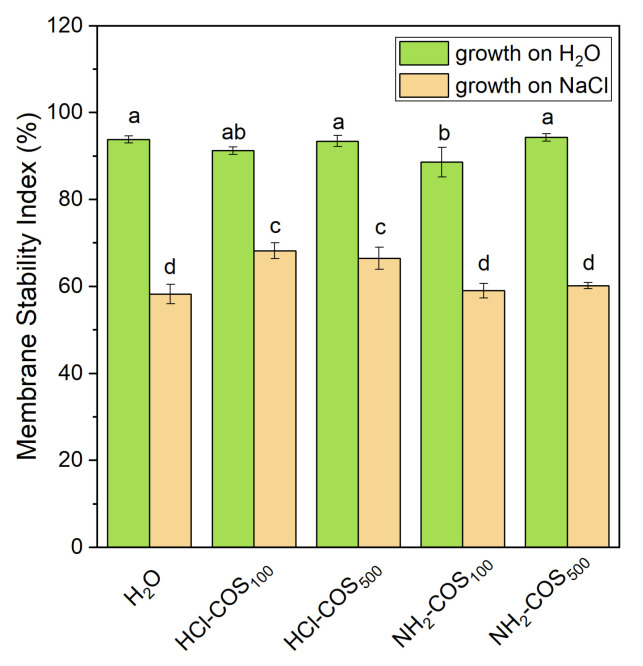
Membrane Stability Index determined for 14-day-old seedlings derived from H_2_O- and COS-primed variants grown in H_2_O or 50 mM NaCl. Values are expressed as means ± SE (*n* = 6). Means with no letters in common differ significantly according to the Duncan’s multiple range test (*p* < 0.05).

To assess the effect of COS-priming on the leaf membrane integrity, the Membrane Stability Index (MSI) was determined. Under non-stress conditions, only the NH_2_-COS_100_ variant showed a slight decrease (≈6%) compared with the H_2_O-primed control (Figure 8). Under salt stress, the MSI in both the H_2_O- and NH_2_-COS-primed plants decreased by approximately 38% relative to the non-stressed H_2_O-primed sample. In contrast, the decline in MSI was less pronounced in the HCl-COS variants, which maintained 14–17% higher values than the H_2_O-primed stressed plants.

### 2.7. Statistical Evaluation of the Individual and Combined COS-Priming and Salt Effects

ANOVA, followed by the Duncan’s multiple range test, was used to evaluate the individual and combined effects of COS-priming (both HCl-COS and NH_2_-COS) and salt stress on the physiological parameters determined in this study. The analysis revealed that continuous exposure to 50 mM NaCl stress affected almost all parameters assessed in 4- and 14-day-old seedlings, including root length, Na^+^ accumulation in roots and leaves, leaf water potential, photosynthetic activity, content of leaf pigments and MSI (Table 3). COS-priming alone significantly affected seed surface roughness, root length and flavonoid content, the non-photochemical dissipation of excitation energy, flavonoid level, and MSI. The evaluation of the F-values obtained for the combination of salt-stress and COS-priming (F_salt×COS_) revealed that many of the parameters remained unaffected, demonstrating the generally protective effect of the utilized COS under salt stress. The photosynthetic parameters Fv/Fo, Vj, DIo/RC, φ(Eo), PI_total_ related to the operation of PSII, the linear electron-transport, and the non-photochemical dissipation of the excess light energy, remained affected in the COS-primed variants under salt stress, although with relatively low F_salt×COS_ values (ranging from 2.70 to 8.05). A notable high F_salt×COS_ value for the MSI (144.11) was revealed, confirming the positive effect of COS-priming on the plant‘s cellular integrity.

## 3. Discussion

In this work, we investigated the effect of seed priming with aminooligochitosan (NH_2_-COS) and oligochitosan hydrochloride (HCl-COS) on pea seed germination, subsequent seedling growth, and photochemistry of photosynthesis under both physiological and salt stress conditions. The rationale for this work stems from the fact that, although the effects of various chitosan and chitosan derivatives have been extensively studied following foliar application, only a limited number of reports have examined seed treatment and priming protocols. Furthermore, to the best of our knowledge, there are no studies integrating detailed characterization of seed surface topology and PSII functionality in the context of COS seed priming. This gap is noteworthy, as seed priming is expected to provide long-term seed/plant protection and thereby preserve overall plant health. Our results demonstrate that HCl-COS and NH_2_-COS, applied as priming agents at concentrations in the range of 100–500 mg/L, had only minor effects on pea plant growth under non-stress conditions. However, under continuous salt stress (50 mM NaCl), both compounds exhibited dose- and type-dependent effects on pea seeds and seedlings. Furthermore, our findings have revealed a generally protective effect of both chitosan derivatives on plants fitness under saline conditions, as evidenced by positive changes in several physiological parameters associated with photosynthetic activity of photosystem II and cellular membrane integrity. In particular, the most pronounced beneficial effects on the primary photosynthetic steps were observed following treatments with HCl-COS_100_ and NH_2_-COS_500_.

### 3.1. Effect of COS on Seed and Plant Fitness in Non-Stress Conditions

The HCl-COS- and NH_2_-COS-priming treatments used in this study induced structural modifications on the pea seed surface, as revealed by AFM imaging (Figure 1). The seed roughness analysis showed that the observed circular structures in HCl-COS_100_, HCl-COS_500_ and NH_2_-COS_500_-primed variants were higher than those in the control H_2_O-primed variant (Table 1). This most likely indicates that at least a fraction of the COS molecules becomes adsorbed onto the seed surface, potentially forming a homogeneous film that does not disturb the overall surface structure, seed integrity, or germination properties (Figure 1, Figures S1 and S2). Furthermore, these observations demonstrate that the two different COS treatments exert distinct, type- and dose-dependent effects on the seat coat, which might result in different physiological consequences.

The negative effect of COS-priming on root length may be interpreted in the light of the findings reported by [36], who discuss the contrasting (positive and negative) effects of different chitosans on root and shoot growth. Specifically, in a number of examples root length was inhibited following chitosan application in hydroponic or nutrient-based systems, with the outcome depending on chitosan type and concentration, plant species, and overall experimental conditions. The suppression of genes related to root elongation, along with the stimulation of genes involved in the auxin biosynthesis pathway, resulting in auxin accumulation in the root meristem and subsequent root elongation [37,38], has been identified as a plausible mechanism underlying the observed root length inhibition. Nevertheless, to the best of our knowledge, the data on root growth following pea seed priming presented in the current work are reported for the first time. In our study, we did not perform genetic analysis but instead explored the potential effect of COS-induced structural changes on the seed coat that might affect the seed germination process. Our data confirm that the negative effect on root length is not caused by physical disruption of the seed, as evidenced by the preserved seed coat integrity. This finding justifies further research on the COS-induced hormonal and metabolic changes. Finally, the stability of total chlorophyll content in plants developed from COS-primed seeds under control conditions indicates that COS treatment does not interfere with basal photosynthetic capacity.

Furthermore, chitosan is well-known to boost the roots defense systems via a “growth-defense trade-off” mechanism that ensures higher resistance later in development [36]. The stimulation of flavonoid biosynthesis by HCl-COS_100_ under non-stress conditions (Figure 7) suggests that at this dose HCl-COS can activate the phenylpropanoid pathway, potentially enhancing the plant’s basal antioxidant capacity [24,39]. This observation, however, requires further experimental verification. The absence of similar effects in other treatments highlights the importance of dose- and compound-specific responses.

### 3.2. Effect of COS on Seed and Plant Fitness in Growth Under 50 mM NaCl Salt Stress

The applied salt stress resulted in root diminution for all COS treatments, but also in improved PSII capacity as compared to the H_2_O-primed control. This strongly suggests that both NH_2_-COS and HCl-COS applied at concentrations of 100 or 500 mg/L trigger the above-mentioned “growth-defense tradeoff” mechanism of pea seeds. This was evident from the chlorophyll fluorescence data presented in Figure 5 and Figure 6, which thus deserves a detailed discussion.

The data in the present study revealed a strong influence of salt stress on the examined JIP parameters in H_2_O-primed control seeds (Figure 5 and Figure 6, and Table 2), indicating impairment of the early steps of the photosynthetic process. The increase in the parameter Vj and the decrease in the Fv/Fo ratio in the H_2_O-variant (Figure 5) suggest changes in the acceptor and donor sides of PSII, respectively [40,41]. It is to be noted that the decrease in the parameter Fv/Fo is a result of an increase in Fo, which indicates the disconnection of the light-harvesting complex from the PSII reaction center, which strongly suggests structural reorganization of the photosynthetic complexes within the membrane. Similar salt-induced changes in the PSII complex have also been reported in previous studies [12,42,43]. Modifications of the PSII acceptor side influence Q_A_ reoxidation and its interaction with plastoquinone [12], leading to limitations in electron transport beyond Q_A_^−^ (φEo). In addition, the decrease in the RC/ABS ratio reveals significant inactivation of the active PSII RC under the applied NaCl stress, as observed previously [44,45]. Prior studies with sorghum also showed that when seedlings were treated with 50 mM NaCl, the chlorophyll content did not change, but changes were observed in the PSII complex. Specifically, changes were found in absorption flux per reaction center and electron transport flux per reaction center [46]. Our data also revealed that salt stress decreased the probability of reduction in the end electron acceptors at the PSI acceptor side (δRo) (Figure 5), which suggests diminished reduction from NADP+ to NADPH via ferredoxin- NADP+ reductase and decreased efficiency of the Calvin–Benson cycle in line with [47]. It has also recently been shown that NaCl inhibits the activity of the Calvin−Benson cycle at least in two different sites [48].

All these salt-induced alterations in PSII resulted in a reduction in the performance indices (PI_ABS_ and PI_total_), as these changes were associated with a stronger impact on the performance of thermal reactions between the intersystem electron carriers [ψ(Eo)/(1 − ψ(Eo))] and on the performance of the primary photochemistry [φ(Po)/(1 − φ(Po))]. The inhibition of the functions of the electron-transport chain under applied salt stress was accompanied by an increase in energy dissipation (DIo/RC), which is a protective mechanism preventing further damage to the photosynthetic apparatus under abiotic stress [49].

Previous works have also revealed that salt stress mainly destroys the OEC, inactivates PSII reaction centers, and blocks the electron flow from Q_A_ to Q_B_ in the photosynthetic electron chain [44,50]. The inhibition of PSII donor side activity is possibly related to the destabilization of the catalytic Mn complex due to salt-induced release of the Mn-stabilizing PsbO protein [51].

A study by [52] demonstrated that salt stress inhibits the activity of the PSII RC in plant leaves by reducing the activity of the OEC at the donor side of PSII and degrading D1 protein on the acceptor side of the PSII. A decrease in the electron transfer rate results in the accumulation of excess electrons from the electron transfer chain and leads to electron leakage, which could increase ROS and exacerbate damage to the PSII RC. Ultimately, it may result in peroxidation or dissociation of thylakoid membranes, i.e., alteration of the thylakoid ultrastructure [53]. A recent study on tomato varieties, characterized by different salt tolerance, demonstrated that salt stress inhibited the activity of both PSII and PSI by suppressing electron transfer efficiency, causing multiple damaged sites on the donor and acceptor sides of PSII, and disturbed energy flow distribution [54]. It is concluded that the salt tolerance mechanism in tomato is attributed to higher carbon assimilation efficiency and sugar accumulation, as well as the ability to protect PSII structures and maintain better PSI electron transfer.

Under salt stress conditions, all COS-primed variants exhibited values of the parameters Fv/Fo, Vj and RC/ABS similar to those of non-stressed H_2_O-primed plants, with a more pronounced effect in the HCl-COS_100_ and NH_2_-COS_500_ variants, suggesting enhanced protection of the PSII complex in these COS-primed plants (Figure 5). Moreover, COS-priming prevents the salt stress-induced decrease in δRo parameter (Figure 5), which suggests better reduction from NADP+ to NADPH and improved efficiency of the Calvin–Benson cycle. In addition, the performance indices (PI_ABS_ and PI_total_) for COS-primed variants were also higher than those for stressed H_2_O-primed plants (Figure 6), thus revealing a preservation of the primary photochemical reactions. This effect may result from the activation of enzymes involved in the light-dependent photosynthetic reactions, which enhances antioxidant protection and helps preserve relative membrane integrity under salt stress [24,55,56]. However, we did not find significant differences in the chlorophyll content under non-stress and salt stress conditions, which shows that the protective effect of COS-priming on photosynthetic parameters is more likely due to structural rearrangements of the photosynthetic thylakoid membranes, which optimize the photosynthetic process—an issue that will be further explored by us in future.

Interestingly, the positive effects observed in PI_ABS_ and PI_total_ parameters were the highest (on average) for the two variants that exhibited the highest *R_rms_* values, i.e., HCl-COS_100_ and NH_2_-COS_500_. This might be due to differences in the internalization and metabolism of the two COS due to their different chemical structures, which requires further detailed studies.

To quantify how well cell membranes of pea leaves maintain their integrity under conditions of COS-priming and salt stress, and their combined effect, here we evaluated the MSI, which reflects the degree of injury of leaf cellular membranes—one of the earliest and most sensitive response to salt stress [11,57,58]. Our data revealed lower stress-induced injury under salinization for both HCl-COS variants, meaning that only HCl-COS-priming exhibited a pronounced protective effect regarding the leaf membranes’ integrity under salt stress.

Under salt stress, the marked decrease in flavonoid content in H_2_O-primed and NH_2_-COS_500_ plants suggests a disruption of secondary metabolism or increased utilization of flavonoids as antioxidants in response to elevated ROS levels [17,19]. The relatively stable flavonoid levels in NH_2_-COS_100_ plants under salt stress (i.e., similar to those recorded under non-stress conditions) point to a partial protective effect at the 100 mg/L concentration, possibly through improved redox homeostasis. In HCl-COS-treated plants, the reduction in flavonoids under salinity, irrespective of concentration, may reflect a metabolic shift toward primary stress responses or an enhanced consumption of these compounds for ROS detoxification [59].

Anthocyanin responses further support the role of COS in modulating stress-related secondary metabolism [60]. While anthocyanin levels remained unchanged under control conditions, their significant reduction under salt stress (especially in H_2_O-primed, HCl-COS_100_, and NH_2_-COS_100_ plants) suggests that salinity suppresses their biosynthesis or accelerates their degradation. Notably, the maintenance of high anthocyanin levels in HCl-COS_500_ and NH_2_-COS_500_ plants under salt-stress (with respect to these treatments under non-stress conditions) may indicate a concentration-dependent protective effect, potentially linked to enhanced stress signaling or stabilization of pigment metabolism at higher COS doses. Given the role of anthocyanins as light filters and antioxidants, their relative preservation in these treatments may contribute to improved photoprotection under saline conditions [18].

In summary, this study provides the first experimental evidences of strong positive effects of seed priming with HCl-COS_100_ and NH_2_-COS_500_ on pea primary photosynthetic reactions under salt stress conditions. Our results strongly suggest that commercially available NH_2_-COS and HCl-COS are effective seed priming agents. These compounds preserve PSII operation and membrane cell integrity, and stimulate the biosynthesis of compounds with protective function under salt stress conditions, thus providing an affordable alternative for seed treatment with beneficial aspects for plant health, although further investigation and verification at the whole-plant level are necessary before any conclusions on whole-plant resilience can be drawn. To understand the exact mechanism(s) of action of these two oligochitosans, evaluation of the genetic, hormonal, metabolic, and antioxidant status of the plants is needed, as well as studies on shoot growth and biomass accumulation to confirm whole-plant tolerance.

## 4. Materials and Methods

### 4.1. Seed Treatment and Germination

For this study, we utilized two low-molecular-weight COS: chitosan hydrochloride (ChiBio, Qingdao, China) with deacetylation degree ≥ 98% and viscosity (1% in water) 20 cps, and aminooligochitosan (Realfine Chemical, Wuxi, China) with deacetylation degree ≥ 80% and viscosity (5% in water) < 15 cps. Both COS were dissolved in distilled water at a concentration of 100 mg/L and 500 mg/L and used fresh solutions for seed priming.

Sterilization of pea seeds with 0.1% KMnO_4_ for 1 min, followed by thorough washing with distilled water, was performed prior to the seed priming treatment. For each priming experiment, batches of 50 pea seeds (*Pisum savitum* L, cv. RAN-1) were soaked in 50 mL of either distilled water (H_2_O-priming) or the two aqueous solutions of chitosan oligosaccharides (COS-priming, denoted as NH_2_-COS_100_, NH_2_-COS_500_, HCl-COS_100_ and HCl-COS_500_ for concentration 100 and 500 mg/L, respectively). The priming procedure was conducted for 8 h under continuous and gentle rotation at 2 rpm and room temperature. Seed imbibition percentage was determined by weighing the dry mass of the seeds before the priming and immediately after 8 h of the incubation period. At that point, the seed coat integrity was also evaluated through the degree of electrolyte leakage from the seeds, estimated by measuring the electrical conductivity of all the priming solutions before and after the treatment (HI-5321 research grade EC/TDS meter, Hanna Instruments, Smithfield, RI, USA). The treated seeds were dried for 14–20 days to their initial weight in a dark and dry place. The priming procedure and all subsequent biological experiments were performed in triplicate.

### 4.2. Seed Surface Properties

Surface investigation of both H_2_O- and COS-primed pea seeds was carried out using atomic force microscopy (MFP-3D, Asylum Research, Oxford Instruments, Santa Barbara, CA, USA). Silicon AFM probes (AC160TS), with a resonance frequency of 300 kHz and a nominal spring constant of 20 N/m, were employed. All measurements were performed in air at room temperature in contact mode on primed seeds that were rehydrated for 3 h in distilled water and consequently dried out. Morphometric characterization, including surface roughness (*R_rms_*) analysis, was conducted using IgorPro 6.37 software. *R_rms_* was calculated according to the following formula:

$$R_{rms}=\sqrt{\frac{1}{N}}\sum_{i=1}^{N}Z_{i}^{2}$$

where *Z_i_* is the height at a given pixel *i* and *N* is the total number of pixels in the image.

### 4.3. Growth Conditions for Control and Stressed Seedlings

Before the start of germination, 25 seeds from each batch of 50 primed seeds were soaked in 50 mM NaCl for a 3-h rehydration period in order to induce salt stress at the earliest stage of plant development and to mimic natural conditions where seeds are exposed to salt stress already at their germination period. The other 25 seeds served as controls and were rehydrated and consequently grown in distilled water. All rehydrated seeds were placed in Petri dishes to germinate on filter paper saturated with tap water (pH = 7, conductivity 80 µS/cm^2^) for control seeds or a 50 mM NaCl solution for stressed seeds and were incubated in darkness. Germination and root length were recorded at 24-h intervals for 4 days. The germinability and synchrony of germination were calculated according to [61].

Young 4-day-old seedlings were transferred for hydroponic growth in containers filled with tap water (for control seedlings) or aqueous solution of 50 mM NaCl (for stressed seedlings), in controlled laboratory conditions, as previously described by us [62]. The plants were grown for a period of 14 days, which was sufficient for proper anatomical development of the third pair of true leaves. The second and third pairs of leaves were used for subsequent analyses.

### 4.4. Leaf Water Potential Measurements

Leaf water potential (ψ_leaf_) was measured *in situ* using a PSY1 leaf psychrometer (ICT International, Armidale, NSW, Australia). For this purpose, the leaf chamber of PSY1 sensor was tightly attached onto the axial surface of the third leaf of intact control and salinity-stressed leaves, after gentle scratching and removal of the cuticular layer to ensure proper vapor equilibration and measurement accuracy. For each individual leaf, the measurement duration was set to 5 min.

### 4.5. Sodium Ion Content in Leaves and Roots

The Na^+^ content was determined by using a portable ion-selective meter L-AQUAtwin (Horiba, Kyoto, Japan). Measurements were performed on supernatant derived from centrifugation of homogenized roots and leaves (second and third pair) of individual plants. Prior to each measurement series, the instrument was calibrated using a two-point calibration procedure with standard solutions of 500 and 2000 ppm Na^+^. Sodium content was expressed as the [Na]^+^/g DM of the homogenized plant material ratio.

### 4.6. Fluorescence Induction Kinetics

Chlorophyll *a* fluorescence induction kinetics was recorded as OJIP transients using a Handy PEA fluorimeter (Hansatech Instruments Ltd., Narborough Road, Pentney, UK). Data acquisition and processing were performed with PEA Plus software (version 1.13). Prior to measurement, leaf samples were dark-adapted for 15 minutes using standard leaf clips to ensure full reopening of PSII RC. Fluorescence induction was initiated with a saturating red actinic pulse of 3200 µmol m^−2^ s^−1^, and the full OJIP transient was recorded. The primary fluorescence values obtained were Fo (minimal fluorescence), Fj (fluorescence at the J-step, ~2 ms), Fi (fluorescence at the I-step, ~20 ms), and Fm (maximal fluorescence). These raw values were used to calculate the updated JIP-test parameters included in this study. The following parameters were calculated according to [34]: F_V_/Fo—indicator of PSII photochemical efficiency and RC openness; Mo is defined as the initial slope of relative variable fluorescence; Mo = 4(F_300μs_ − Fo)/(Fm − Fo); Vj = (Fj − Fo)/(Fm − Fo)—relative variable fluorescence at the J-step indicating the accumulation of Q_A_^−^; φEo = Fv/Fm × ψEo—quantum yield of electron transport beyond Q_A_^−^; ψEo = (1 − Vj)—probability that a trapped exciton moves an electron into the electron transport chain beyond Q_A_^−^; δRo = (1 − Vi)/(1 − Vj)—probability that an electron is transferred from the intersystem chain to PSI end acceptors; φPo = Fv/Fm—maximum quantum yield for primary photochemistry; ABS/RC = Mo × (1/Vj) × (1/φPo); RC/ABS or (1/ABS/RC)—density of active PSII RC per absorbed energy; DIo/RC = ABS/RC(1 − φPo)—energy dissipation per reaction center; PI_ABS_—performance index on an absorption basis and PSII efficiency; PI_total_—total performance index including PSI contribution [63]. The two performance indices were calculated according to the following equations [64]:

PI_ABS_ = [γRC2/(1 − γRC2) = RC/ABS] × [φPo/(1 − φPo)] × [ψEo/(1 − ψEo)]

and

PI_total_ = PI_ABS_ × [(δRo/(1 − δRo)]

### 4.7. Measurements of Leaf Pigments

Total chlorophyll, flavonols and anthocyanin contents were non-destructively determined using a multi-pigment meter (MPM-100, Opti-Sciences Ins., Hudson, NH, USA).

### 4.8. Determination of Membrane Stability Index

Membrane integrity of pea leaves was assessed by calculating the membrane stability index (MSI) using the following equation: MSI (%) = [1 − (EC1/EC2)] × 100, where EC1 and EC2 are the measured conductivities of leaf material submerged in distilled water after incubation in a water bath at 40 °C for 30 min and after heating at 100 °C, respectively, for 15 min as in [57,58].

### 4.9. Graphical Representation and Statistical Evaluation

Data were graphically presented using Origin 2018 (Origin Lab., Northampton, MA, USA). The comparisons between the responses to salt and chitosan treatments were made using a two-way ANOVA, followed by the Duncan’s multiple range test (*p* < 0.05). R-4.5.2 (R-project, https://cran.r-project.org/, R Foundation, Vienna, Austria) software with *agricolae* package (https://cran.r-project.org/package=agricolae (accessed on 1 April 2026)) was used for the calculations.

## Figures and Tables

**Table 1:** Seed surface roughness (***R_rms_***) values determined for H_2_O- and COS-primed variants.

Treatments	*R_rms_* (nm)
H_2_O	266 ± 31 ^c^
HCl-COS_100_	404 ± 22 ^ab^
HCl-COS_500_	339 ± 30 ^b^
NH_2_-COS_100_	264 ± 30 ^c^
NH_2_-COS_500_	421 ± 18 ^a^

Values are expressed as means ± SE (*n* = 10–16 images for each variant). Means with no letters in common differ significantly according to the Duncan’s multiple range test (*p* < 0.05).

**Table 2:** Components of the performance indices PI_ABS_ and PI_total_ estimated for 14-day-old seedlings derived from H_2_O- and COS-primed seeds grown in H_2_O or 50 mM NaCl.

Treatments	γ(RC)/((1 − γ(RC))	φ(Po)/((1 − φ(Po))	ψ(Eo)/(1 − ψ(Eo))	δ(Ro)/(1 − δ(Ro))
H_2_O	0.411 ± 0.005 ^a^	5.698 ± 0.061 ^a^	0.999 ± 0.018 ^a^	0.729 ± 0.028 ^ab^
HCl-COS_100_	0.402 ± 0.004 ^ab^	5.538 ± 0.056 ^b^	0.938 ± 0.009 ^b^	0.731 ± 0.022 ^ab^
HCl-COS_500_	0.411 ± 0.005 ^a^	5.540 ± 0.087 ^ab^	0.979 ± 0.018 ^a^	0.789 ± 0.026 ^a^
NH_2_-COS_100_	0.410 ± 0.004 ^a^	5.419 ± 0.069 ^ab^	0.983 ± 0.011 ^a^	0.765 ± 0.027 ^ab^
NH_2_-COS_500_	0.409 ± 0.004 ^a^	5.585 ± 0.062 ^ab^	0.987 ± 0.014 ^a^	0.775 ± 0.023 ^ab^
NaCl	0.338 ± 0.012 ^d^	3.797 ± 0.273 ^d^	0.769 ± 0.041 ^d^	0.655 ± 0.029 ^c^
HCl-COS_100_ + NaCl	0.396 ± 0.006 ^bc^	4.928 ± 0.148 ^bc^	0.898 ± 0.026 ^bc^	0.718 ± 0.041 ^b^
HCl-COS_500_ + NaCl	0.367 ± 0.008 ^cd^	4.596 ± 0.209 ^c^	0.877 ± 0.058 ^c^	0.674 ± 0.034 ^bc^
NH_2_-COS_100_ + NaCl	0.383 ± 0.014 ^bc^	4.399 ± 0.418 ^c^	0.792 ± 0.085 ^d^	0.769 ± 0.054 ^ab^
NH_2_-COS_500_ + NaCl	0.389 ± 0.008 ^bc^	4.858 ± 0.211 ^bc^	0.901 ± 0.038 ^bc^	0.743 ± 0.039 ^ab^

Values are expressed as means ± SE (*n* = 20). Means with no letters in common differ significantly according to the Duncan’s multiple range test (*p* < 0.05).

**Table 3:** Summary of one-way and two-way ANOVA for the effects of salt (F_salt_), COS-priming of seeds (F_COS_), and their interaction (F_salt×COS_) on seed structural and plant physiological traits *in Pisum sativum*.

Trait	F_salt_	F_COS_	F_salt×COS_
*R_rms_*		11.92 ***	
Imbibition		2.48	
Conductivity		2.47	
Germination	0.48	0.76	0.41
Synchrony	0.12	0.33	0.23
Root length	62.33 ***	6.33 ***	2.81 *
Leaf water potential	40.58 ***	0.27	0.36
Root Na^+^ content	290.15 ***	1.62	1.78
Leaves Na^+^ content	206.59 ***	0.39	0.31
Fv/Fo	92.87 ***	2.63 *	6.05 ***
Vj	17.60 ***	1.36	2.70 *
DIo/RC	55.80 ***	3.62 **	8.05 ***
φ(Eo)	32.60 ***	1.57	3.74 **
RC/ABS	27.44 ***	1.20	0.93
δ(Ro)	33.54 ***	1.40	0.21
PI_ABS_	34.13 ***	2.37	2.05
PI_total_	71.02 ***	1.83	3.81 **
Chlorophyll	8.58 **	0.63	0.34
Flavonoids	93.29 ***	2.80 *	2.10
Anthocyanins	65.16 ***	0.78	2.07
Membrane Stability Index	430.64 ***	4.91 *	144.11 ***

*** indicates 99.9% significance, ** indicates 99% significance, * indicates 95% significance.

## Data Availability

All data are contained within the manuscript.

## Supplementary Materials

The following supporting information can be downloaded at: https://www.mdpi.com/article/10.3390/ijms27104498/s1.

## Abbreviations

The following abbreviations are used in this manuscript:

AFM	atomic force microscopy
COS	chitooligosaccarides
DM	dry mass
MSI	Membrane Stability Index
NH_2_-COS_100_	aminooligochitosan in concentration 100 mg/L
NH_2_-COS_500_	aminooligochitosan in concentration 500 mg/L
HCl-COS_100_	oligochitosan hydrochloride in concentration 100 mg/L
HCl-COS_500_	oligochitosan hydrochloride in concentration 500 mg/L
OEC	oxygen-evolving complex
PSI	photosystem I
PSII	photosystem II
RC	reaction center
ROS	reactive oxygen species
Fv/Fo	ratio of quantum yields of photochemical and concurrent non-photochemical processes
Vj	relative variable fluorescence at the J step, indicating the proportion of closed photosystem II reaction centers
δRo	efficiency/probability of reduction in the end electron acceptors at the photosystem I acceptor side
RC/ABS	density of the active reaction centers
φEo	quantum yield of electron transport beyond Q_A_
DIo/RC	quantum yield of energy dissipation in the form of heat and fluorescence at the reaction center level
R_rms_	seed surface roughness
PI_ABS_	performance index on absorption basis
PI_total_	total performance index
Ψ_leaf_	leaf water potential
