# Supplementary material for: Chitooligosaccharide Seed Priming Enhances Photosynthetic Efficiency in Pea (Pisum sativum) Under Salinity
Source: Int J Mol Sci. 2026 May 18;27(10):4498. doi: 10.3390/ijms27104498 (PMC13207665; doi:10.3390/ijms27104498)
Supplement: Supplementary file 1 [file ijms-27-04498-s001.zip › ijms-4295662-supplementary.pdf]

Supplementary Materials

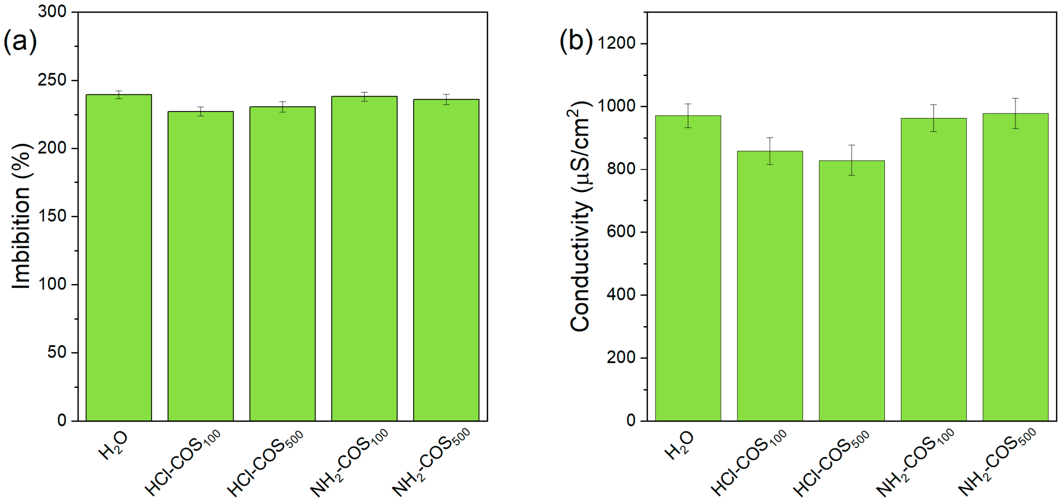

Figure S1. Imbibition (**a**) and conductivity (**b**) values determined for seeds incubated for 8h in water (H_2O-priming) or in solutions of the two chitooligosaccharides at concentrations of 100 mg/L (HCl-COS_{100}, NH_2-COS_{100}) and 500 mg/L (HCl-COS_{500}, NH_2-COS_{500}). Values are expressed as means ± SE (n = 3–5). One-way ANOVA followed by Duncan's multiple range test were applied, and no statistically significant differences were found ($p < 0.05$).

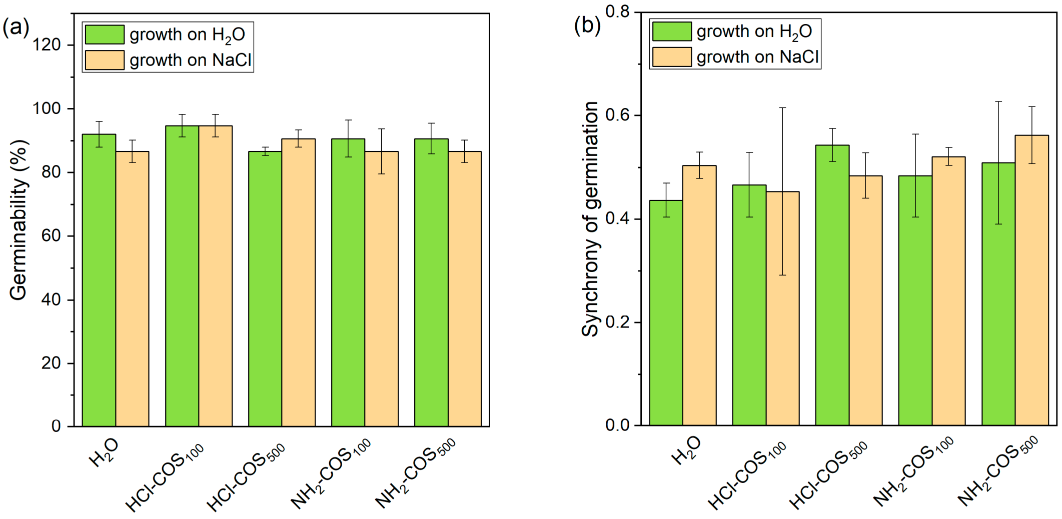

Figure S2. Germinability (**a**) and germination synchrony (**b**) of H_2O- and COS-primed seeds, grown under control conditions or in 50 mM NaCl. Values are expressed as means ± SE (n = 3). Two-way ANOVA followed by Duncan's multiple range test were applied, and no statistically significant differences were found ($p < 0.05$).

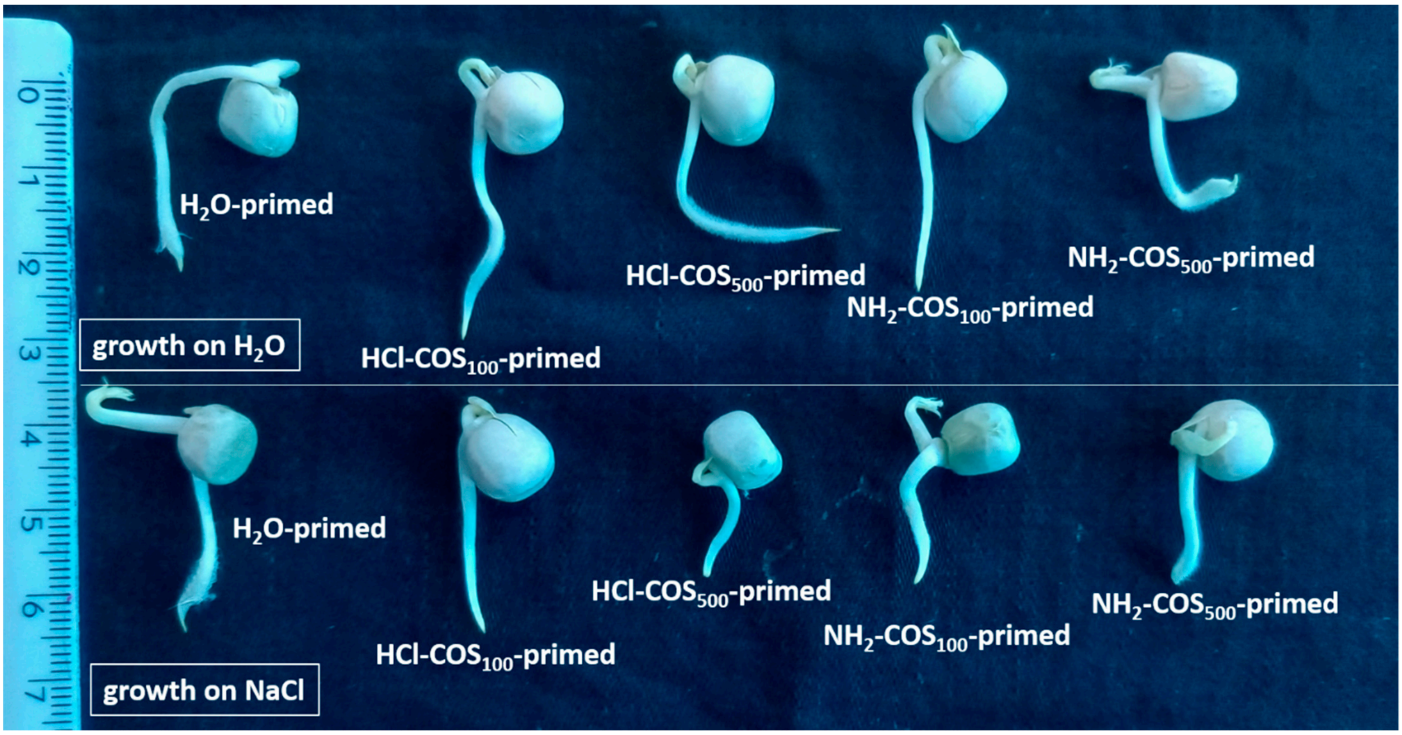

Figure S3. Representative images of roots developed from seeds of 4-day-old control and salt stressed H_2O- and COS-primed variants.

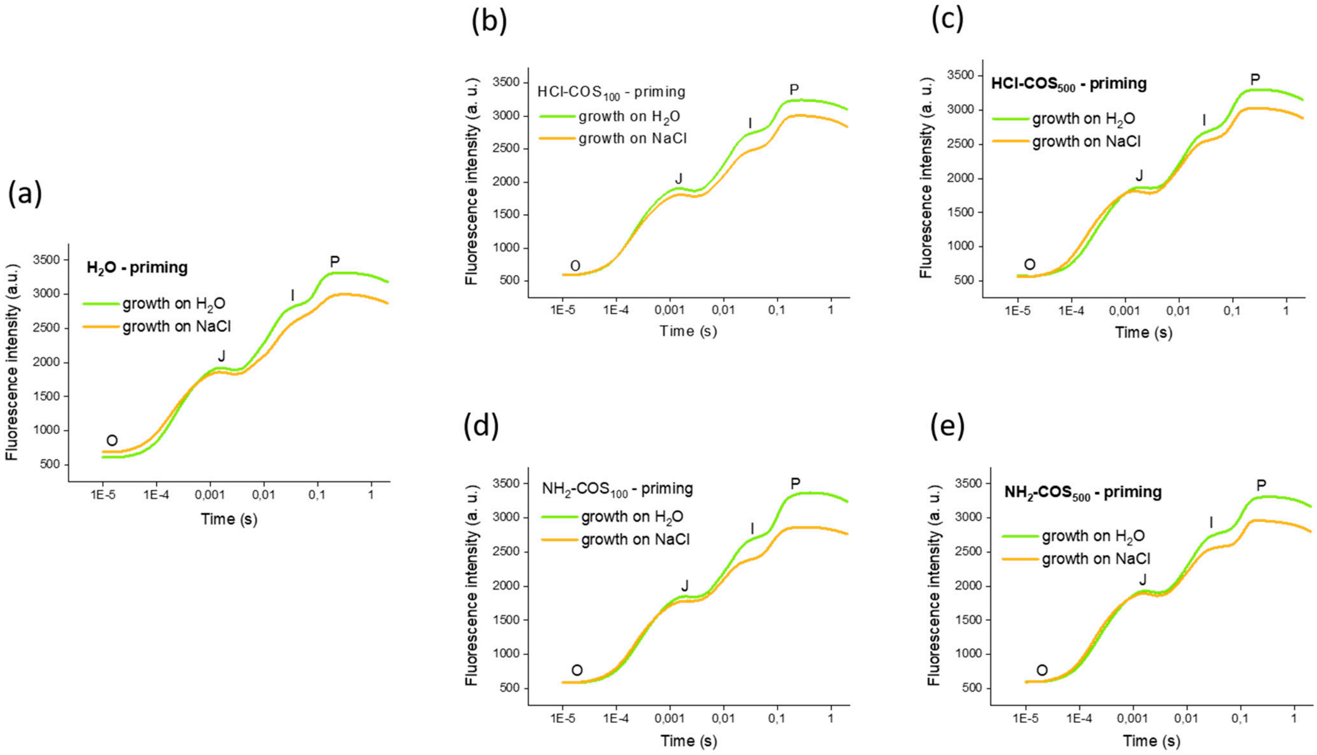

Figure S4. Representative OJIP curves recorded for 14-day-old control and salt stressed H_2O- and COS-primed variants.
